# Impact of Self-Assembled Monolayer Design and Electrochemical Factors on Impedance-Based Biosensing

**DOI:** 10.3390/s20082246

**Published:** 2020-04-16

**Authors:** Michael C. Brothers, David Moore, Michael St. Lawrence, Jonathan Harris, Ronald M. Joseph, Erin Ratcliff, Oscar N. Ruiz, Nicholas Glavin, Steve S. Kim

**Affiliations:** 1 711th Human Performance Wing, Air Force Research Laboratory, Wright-Patterson AFB, OH 45433, USA; mbrothers@ues.com (M.C.B.);; 2 UES Inc., Dayton 45432, OH, USA; 3 Materials and Manufacturing Directorate, Air Force Research Laboratory, Wright-Patterson AFB, OH 45433, USA; 4 Aerospace Systems Directorate, Air Force Research Laboratory, Wright-Patterson AFB, OH 45433, USA; 5 University of Dayton Research Institute, Dayton, OH 45469, USA; 6 Department of Chemical Engineering, University of Arizona, Tucson, AZ 85721, USA; 7 Department of Materials Science and Engineering, University of Arizona, Tucson, AZ 85721, USA

**Keywords:** impedance biosensor, protein sensor, electrochemical impedance spectroscopy, self-assembled monolayer

## Abstract

Real-time sensing of proteins, especially in wearable devices, remains a substantial challenge due to the need to convert a binding event into a measurable signal that is compatible with the chosen analytical instrumentation. Impedance spectroscopy enables real-time detection via either measuring electrostatic interactions or electron transfer reactions while simultaneously being amenable to miniaturization for integration into wearable form-factors. To create a more robust methodology for optimizing impedance-based sensors, additional fundamental studies exploring components influencing the design and implementation of these sensors are needed. This investigation addresses a sub-set of these issues by combining optical and electrochemical characterization to validate impedance-based sensor performance as a function of (1) biorecognition element density, (2) self-assembled monolayer chain length, (3) self-assembled monolayer charge density, (4) the electrochemical sensing mechanism and (5) the redox reporter selection. Using a pre-existing lysozyme aptamer and lysozyme analyte combination, we demonstrate a number of design criteria to advance the state-of-the-art in protein sensing. For this model system we demonstrated the following: First, denser self-assembled monolayers yielded substantially improved sensing results. Second, self-assembled monolayer composition, including both thickness and charge density, changed the observed peak position and peak current. Third, single frequency measurements, while less informative, can be optimized to replace multi-frequency measurements and in some cases (such as that with zwitterionic self-assembled monolayers) are preferred. Finally, various redox reporters traditionally not used in impedance sensing should be further explored. Collectively, these results can help limit bottlenecks associated with device development, enabling realization of next-generation impedance-based biosensing with customize sensor design for the specific application.

## 1. Introduction

Substantial advances have been made in pseudo-real-time sensors for biochemical monitoring [1]. However, implementation of these sensors on the government/commercial market has been limited to enzymatic-based sensors that generate redox-active byproducts (i.e., glucose oxidation producing NADH/FADH_2_) [2] and ionophore-based sensors [3] that use ion separation to generate a measurable potential. Even within the literature, few sensing platforms exist for real-time measurement of protein concentrations. The over-arching challenge is the need to convert the binding event into an observable signal. Most commonly, this is done via detecting the changes in electrostatic interactions between analytes and biorecognition elements (BREs) in real-time. Widely applied detection methods monitor the thickness of biomolecules on a substrate using optical (i.e., surface plasmon resonance) [4,5,6], guided mode resonance [7], surface-enhanced Raman spectroscopy [8,9,10] or electrochemical means such as direct oxidation of the tyrosines in proteins [11] or impedance spectroscopy [12]. Of these platforms, electrochemical platforms have the greatest promise for miniaturization and implementation in wearable devices.

Impedance sensors have been deployed for a variety of protein targets, using a frequency sweep at a fixed potential (electrochemical impedance spectroscopy, EIS) [13,14,15] as well as a voltage sweep at a fixed frequency (differential pulse voltammetry (DPV) or square-wave voltammetry (SWV)) [16]. However, implementation and optimization of these sensors remains challenging due to non-specific binding, sensor drift and other factors [17] for all impedimetric sensors, particularly those using EIS or SWV measurements, with either the redox probe in solution or immobilized onto a BRE. 

**Figure 1:**
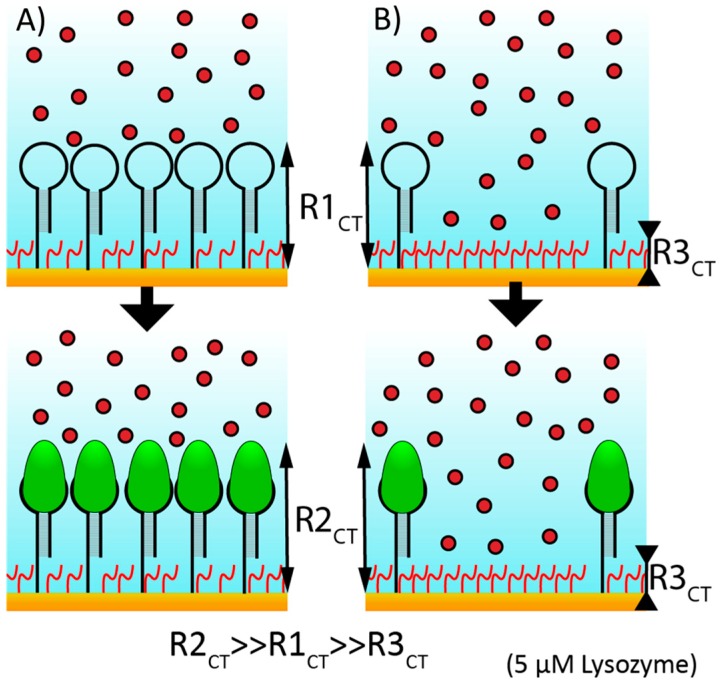
Schematic of electrochemical impedance spectroscopy (EIS) sensing using lysozyme aptamer (loop) and lysozyme antigen (green oval, bottom panel) demonstrates why in this instance only high density self-assembled monolayers (SAMs) (**A**) and not low density SAMs (**B**) are expected to have substantial changes in R_CT_, as they occlude ferricyanide redox reporters (red circles) from the surface.

Electrochemical impedance offers resolution of both energy and/or frequency domains, facilitating detection of specific redox activities and/or capacitive changes with binding events [18]. Particularly, the latter is critical for non-redox active biomarkers, especially in the absence of a redox reporter. In short, a binding event triggers a change in the self-assembled monolayer (SAM) thickness, thus altering both the real and imaginary impedance. One methodology to enhance the sensitivity and robustness includes using impedance-based sensors that have a reporter-tag in solution; these have the substantial drawback of requiring the addition of reagent but the benefit of being easily applied. In theory, a binding event that changes the thickness of the SAM and thus occludes the reporter tag, should result in a change in impedance that can be measured and monitored electrochemically (Figure 1).

An alternative approach has been developed to make this platform reagentless [19]. However, instead of monitoring SAM thickness, reagentless sensing relies on monitoring changes in impedance indicative of conformational changes of the BRE/SAM. In short, a reporter tag is attached directly to the BRE and/or to the linker-group, where a change in conformation of the BRE, triggered upon binding of the analyte to the aptamer, causes a change in location of the redox reporter and thus the resistance to charge transfer (R_CT_). While this approach has been demonstrated effective in complex biological mixtures [20], it also often requires substantial engineering of the aptamer, including truncation of the aptamer sequence and/or placement of the redox probe in a different location to allow for a binding event to trigger a substantial conformational change that leads to a change in current [21]. This technique has most notably been demonstrated only for a select number of targets, primarily small molecules. Therefore, especially for candidate BRE screening, we focused on optimization of the platform with the reagent-added system with the knowledge that some of the lessons learned could also apply to the other platforms, including reagentless impedimetric detection. 

Impedance sensor performance relies on multiple factors that must be addressed in order to properly screen the suitability of a BRE for sensing on an immobilized substrate. First, it has been demonstrated that the ratio of blocking molecules to aptamers can impact sensor performance [22]. However, no quantitative measurement has been performed to accurately quantify the relationship between this ratio and the observed thickness of the SAM, and then to directly compare this to sensor performance to provide insight into the mechanism of impedimetric sensing. Second, while 6-mercapto-1-hexanol (MCH) is the most common SAM used in the literature, especially for protein detection, other SAMs have been demonstrated in these platforms, primarily for DNA detection. Most notably SAMs with varying backbone composition include either modifying the hydrocarbon chain length [23,24,25] or incorporation of polymeric, hydrophilic backbones [26,27]. Additionally, SAMs have been incorporated with diverse functional groups, including those terminated with carboxylates [25], amines [27], zwitterionic phosphocholine head-groups [28] or bivalent hexanedithiol [29]. However, a more systematic understanding of how these factors influence (1) passivation and voltage drop, (2) anti-fouling properties and (3) sensor performance using impedance spectroscopy is lacking.

As an additional parameter to consider, there are multiple redox reporters known in the literature that have properties impacting EIS and SWV measurements. It has been demonstrated that the redox reporters yield more stable sensors when their redox potentials are closer to 0 V vs. SHE (more spontaneous reaction) [30]. Additionally, desorption of cyanide from ferricyanide is known to cause slow etching of gold, degrading the electrodes over time [31]. However, there is a trade-off in performance by moving to other redox reporters that might have more spontaneous redox potentials. Most relevantly, while ferricyanide is an inorganic salt and thus should not perturb protein or aptamer structure, alternative redox couples are organic molecules exhibiting conjugated rings, such as methylene blue, viologen or ferrocene. These alternative redox couples are more hydrophobic and presumably might intercalate with DNA [32] and/or the hydrophobic regions of proteins. 

Therefore, a broader study looking at protein aptasensing using a model system will provide insight and guidance into factors that should be explored for implementation of real-time protein biosensing. Also, an improved understanding of the key factors in biosensing will lead to both an effective screening of candidate BREs as well as an ability to tune the biofunctionalization of sensors, collectively contributing to achieving advanced electrochemical sensing platforms. 

## 2. Materials and Methods

### 2.1. Chemicals

All chemicals used in this study were purchased from Sigma Aldrich unless specified otherwise. All chemicals purchased were used without further purification. Blocking agents for the SAMs included the following hydroxyl terminated molecules—(1) β-mercaptoethanol (2C), (2) 6-mercapto-1-hexanol (6C), (3) 11-mercapto-1-undecanol (11C), as well as the zwitterionic (1) cysteine (2C +/−), (2) 1:1 ratio of (6-mercaptohexyl)trimethylammonium bromide (MTAB) and 6-mercaptohexanoic acid (6C +/−) and (3) 1:1 ratio of 8-mercaptooctanoic acid and 8-amino-1-octanethiol (8C +/−). All SAM reagents were purchased from Sigma Aldrich except for MTAB, which was synthesized as described later in the Methods section. 

### 2.2. Preparation of Biological Buffers

The experiments were performed in 1X HEPES buffered saline at pH 7.4 (HBS). The recipe used resulted in a buffer with the following final components—(1) 50 mM HEPES, (2) 10 mM magnesium chloride, (3) 100 mM sodium chloride, (4) sodium azide (0.01% w/v). For impedance measurements, either 5 mM potassium ferricyanide and 5 mM potassium ferrocyanide were added or 100 µM of methylene blue as a redox reporter. 

### 2.3. Preparation of Gold Electrodes

For electrochemical measurements, gold rod electrodes were acquired from CH Instruments (2 mM diameter). Electrode cleaning has been described previously [33]. In short, the electrodes were polished as prescribed using a microfiber polishing pad and 0.05 µm alumina (CH Instruments). Post-polishing, they were sonicated briefly in 70% ethanol, followed by running 40 cyclic voltammograms in 0.1M K_2_SO_4_ at pH 11 (−0.4 V to −1.8 V) followed by 40 cyclic voltammograms at pH 3 in 0.1 M K_2_SO_4_ (0.4 V to 1.8 V). The alkaline and acidic electrochemical cleaning steps were repeated until less than 1% change in the gold reduction peak height (~0.8 V vs. AgCl) was observed during the acidic electrochemical cleaning step. 

For ellipsometry studies, silica wafers were sputter coated with a titanium seed layer (~10 nm thick) followed by gold (~100 nm thickness). The wafer was then diced into 1 cm × 1 cm squares. The electrodes were sonicated briefly in ethanol and air-dried prior to use to remove residual contaminants. 

### 2.4. Preparation of Aptamer Biorecognition Elements

A lysozyme aptamer from literature with a 6-carbon thiol appended to the 5’ end was purchased from IDT with the following sequence—5’-SH-C_6_-AGCAGCACAG AGGTCAGATG GCAGGTAAGC AGGCGGCTCA CAAAACCATT CGCATGCGGC-3’ [34]. The aptamer was re-suspended into 1 × HBS, diluted to a concentration of 1 µM and mixed with the desired amount/concentration of blocking reagent. To re-fold the aptamers, the solution was then heated to 95 °C for 10 min using a dry bath and let slowly cool by turning off the temperature of the dry bath to below 40 °C. 

### 2.5. Biofunctionalization of Sensors

Gold electrodes were placed in aptamer-containing solutions, either capped or sealed via parafilm and let soak overnight at 37 °C in a dry bath. The electrodes were removed, rinsed in 1X HBS and placed in either 1 mM 6-mercapto-1-hexanol (MCH) or 100 µM zwitterionic SAM for ~15 min to remove hydrophobically absorbed aptamers and to improve electrode passivation.

### 2.6. Ellipsometry Measurements

Ellipsometry measurements were taken with a JA Woollam VASE Ellipsometer on the diced silica wafers coated with gold (see *Preparation of Gold Electrodes*). Measurements were taken only between 270 and 800 nm, as initial measurements of a broader range demonstrated that other wavelengths were unnecessary. Ellipsometry on a blank gold substrate was first performed as a background. A 100% MCH SAM was then deposited on that same substrate and fit to a Cauchy layer, using the weak Lorentz peaks around 368 nm and 508 nm. This experimental data was normalized to the literature thickness of 1 nm, similar to what has been demonstrated previously [35]. Fresh gold substrates exposed to 1 µM aptamer and varying concentrations of MCH were then measured and fit to a Cauchy layer for a total thickness. Each experiment was repeated on three separate samples.

### 2.7. Synthesis of (6-Mercaptohexyl) Trimethylammonium Bromide (MTAB)

Synthesis of MTAB was performed similar to as has been done previously [36]. A typical procedure is as follows (Figure S1)—27.7 g (0.114 mol) of 1,6-dibromohexane and 4.68 g (0.041 mol) of potassium thioacetate were added to a one-neck round-bottom flask (RBF) with a magnetic stirrer and dissolved in 120 mL (27.0% solids) of acetonitrile. The solution was stirred for 12 h at 90 °C. The mixture was filtered and then the acetonitrile was removed in vacuo. The resulting yellow solution was purified via column chromatography to produce 1-[(6-bromopentyl) sulfanyl]ethan-1-one (C1).

This compound was then used as the starting product to synthesize [6-(acetylsulfanyl) hexyl]-N,N,N-trimethylazanium bromide Synthesis (C2). A typical procedure is as follows—5.314 g (0.023 mol) of C1 and 93 mL of tetrahydrofuran (THF) added to a RBF and chilled (dry ice + isopropyl alcohol). 12.89 g (0.218 mol) of trimethylamine was bubbled through the reaction solution and then the solution was allowed to warm up to room temperature. The solution was stirred for 7 days. After 7 days, the precipitate was filtered, washed with THF and dried *in vacuo*. This reagent was then used as the starting product of the next reaction to produce MTAB. In short, 0.9554 g (3.4 mmol) of C2 was added to a RBF was 2 mL HBr and 21 mL ethanol. The reaction was capped with nitrogen gas and stirred for 2 days at room temperature. The ethanol was removed *in vacuo* and the yellow product was stored in a vial and used without further purification. ^1^H NMR was used to confirm MTAB structure (Figure S1).

### 2.8. Electrochemical Characterization of SAMs

Pure SAMs were created by soaking 1 mM of one of six blocking groups O/N in 1X HBS containing 1 M NaCl (to improve packing density by mitigating electrostatic repulsions) onto gold rod electrodes. The electrodes were then placed into a standard 3-electrode cell containing 1X HBS, using a silver/silver-chloride reference electrode (CH Instruments) and a platinum coated titanium rod (EDAQ) as a counter-electrode. A Gamry 600 Potentiostat was used to electrochemically characterize SAMs, probing both peak potential and peak current at 500 Hz, 20 mV amplitude. The sweep range was individualized for each sample to minimize SAM damage from application of excessive oxidation (Range—0 V to 0.1 V past the observed peak). After initial acquisition, 100 µM bovine serum albumin (BSA) was added (solubilized in 1×HBS with ferricyanide) to the electrochemical cell. The solution was mixed and allowed to incubate for 10 min before running an additional voltammogram to determine the impact of the fouling agent on the electrochemical platform. 

### 2.9. Titrations of Lysozyme for Sensor Testing

For all experiments, up to 6 electrodes were placed in a custom jig (Figure S2) to enable near-simultaneous testing of electrodes. 10 mL of redox-reporter containing buffer was added to submerge the electrodes. A script was then set-up to allow for electrochemical measurements at discrete time-points (~10 min apart) followed by mixing to prevent localized depletion of the redox reporter. 

For all titrations, the same addition protocol was used. A stock concentration of 200 µM lysozyme was created by dissolving crystalline lysozyme powder into buffer containing the redox reporter. Serial 10 and 100 × dilutions were used so that the volume added to the jig was greater than 5 µL and less than 1 mL for each addition. We added five serial additions of lysozyme, most commonly resulting in addition of a total of 24 nM, 120 nM, 600 nM, 3 µM and 15 µM of lysozyme to the bulk solution, immediately followed by mixing and incubation for ~10 min before acquiring the next electrochemical measurement. 

### 2.10. Electrochemical Measurements for Titrations of Lysozyme

Measurements were acquired on a PalmSens3 using the MUX8 multiplexer with a common reference and a common ground. For EIS, the direct-current offset (DC offset) was placed at the redox potential of the redox reporter, determined through identifying the voltage at which the impedance reached a minimum while sweeping voltage at a frequency of 100 Hz (~0.2 V vs. Ag/AgCl for ferricyanide). EIS spectra were then acquired for each channel (up to 6 channels per experiment) with an AC_RMS_ amplitude of 5 mV from 5000 Hz to 1 Hz. Analysis of each spectra was performed using software provided by Palmsens through fitting to a modified Randle’s cell as done previously (Figure S3) [37,38]. The resistance to charge transfer (R_CT_) was recorded and further analyzed for each electrode in each condition. 

Square wave voltammetry (SWV), electrochemical voltammograms were acquired by sweeping potential at 10 Hz and 20 mV AC amplitude from 0.05 V to 0.3 V, with a step-size of 1 mV when using ferricyanide redox reporters (Figure S4). When methylene blue was the redox reporter, the amplitude was changed to 10 mV and the scan was changed to sample from −0.15 to 0 V. Each spectrum had the observed peak current reported. 

### 2.11. Analysis of Electrochemical Data

Each measurement (R_CT_ for EIS or peak current for SWV) was recorded and normalized to the final blank before the titration. In order to compensate for sensor drift, at least 3 blanks were acquired before each experiment. The difference between the last two blanks was recorded as the drift correction factor (DCF) for that electrode and was applied to each subsequent measurement in a linear fashion (addition of 1xDCF for the first measurement, 2×DCF for the second measurement, etc.). These values were then divided by the R_CT_ value or peak current for the last blank, providing normalized R_CT_/peak current values. These data were then plotted, including the standard deviations (error bars included when n > 3 to report the standard deviation). Additionally, we ran “t-Test—Two-samples assuming unequal variances” using Microsoft Excel to determine the probability the data outputs came from the same data sets. *p*-values of <0.05 were reported with a * on graphs, <0.01 with ** and <0.001 with ***.

## 3. Results

Impedance-based aptasensors are known to be influenced by factors including but not limited to electrode geometry, material selection (i.e., gold versus graphene), biofunctionalization (i.e., SAM density, SAM composition, electrode material choice), as well as those external to the sensor (i.e., pH, salinity [38] and other biomolecules [20]) [1]. To limit the scope of this paper, we decided to focus on the interplay between biofunctionalization and electrochemical sensing and how both impact sensor response. In particular, we focused on five key factors using either a model biosensor with a lysozyme aptamer to detect lysozyme and/or characterization methodologies to determine how they influenced/impacted electrochemical sensing—(1) BRE density, (2) SAM chain length, (3) electrochemical sensing mechanism, (4) SAM charge density and (5) redox reporter selection. The BRE density has been demonstrated to significantly impact sensor response through varying the number of blocking groups to BREs for the SAM [22,39]. However, the blocking group to aptamer ratio has not been directly linked to a measured SAM thickness yet. Various SAMs have been used in the literature for a multitude of sensor platforms, with stability and anti-fouling properties being selected for select model SAMs [29,40,41]; however, a more comprehensive discussion at the influence on both non-specific binding and passivation is lacking as well. Both EIS and SWV have been used in the literature when redox reporters are both in solution and added to the surface but none have comprehensively provided the resulting sensor performance yet. Finally, while ferricyanide is the standard redox reporter, to-date, few tested sensor performance with a redox reporter other than ferricyanide for protein aptasensors. Therefore, using a combination of our model system in conjunction with additional experiments to further characterize the surface and identify critical properties that impact electrochemical sensing, we begin to address some of the above gaps in impedance-based aptasensing. The results of our findings are summarized in the discussion session in Table 1.

### 3.1. Impact of BRE Density on Sensing Performance

**Figure 2:**
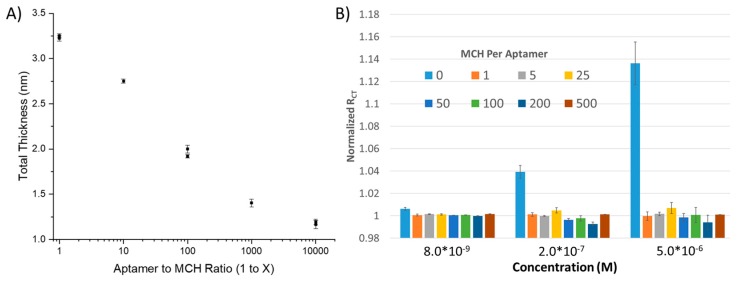
(**A**) Influence of Aptamer to 6-mercapto-1-hexanol (MCH) ratio to surface thickness measured using ellipsometry demonstrates efficacy of arraying MCH concentration to test various densities. (**B**) The lysozyme sensor has a substantially greater response only at the greatest SAM density.

Using chronocoulometry, the density of the aptamers on the surface has previously been demonstrated to be impacted by the MCH to aptamer ratio [42]. However, quantification of the coverage of the BRE on the surface by directly measuring SAM thickness is yet to be explored. Therefore, we took gold-coated silica wafers and placed them overnight in solutions containing 1 µM aptamer to varying concentrations of MCH, with the hypothesis that as the concentration of MCH increases, the number of aptamers per area decreases, as does the apparent film thickness (Figure 2A). Using ellipsometry, we were able to observe what appears to be an exponential decay in film thickness as a function of the MCH to Aptamer ratio. The film thickness appears to have an asymptote at ~1.0 nM, which is the nominal thickness of a MCH SAM (~1.1 Å per bond for 9 bonds). At maximum SAM density, we observe a 3.25 nm thick SAM, which is between~50%–80% of the thickness for an ideally packed SAM; a perfectly globular aptamer will be expected to have a hydrodynamic radius of 2 nm, while a similar sized aptamer form the literature (54 nt) had an experimentally determined hydrodynamic radius of ~3 nm [43]. This deviation from the hydrodynamic diameter can be explained by imperfect packing of aptamers. We would expect nearest-neighbor electrostatic repulsions to result in space between aptamers; additionally, aptamers do not inherently form spherical objects and thus will lead to additional steric repulsions, further reducing packing density. The main implication of this finding for electrochemical sensor development is that sampling of SAM density as a function of SAM thickness is most critical at the lowest concentrations of MCH to aptamer ratio due to the exponential nature of the reduction of film thickness that correlates with an increase in the number of MCH blocking groups. 

To explore this further, we tested the impact of BRE density on aptasensor response using EIS. EIS, through the use of multiple frequencies, has the benefit of enabling separation and calculation of each and every component of the electrochemical cell, including (1) solution resistance, (2) double layer capacitance, (3) R_CT_ (i.e., from the redox reporter) and (4) diffusion effects (Figure S3). However, the downside to this method is both the complexity of instrumentation and circuitry needed to run the assay as well as the time to run each series of scans. Baseline values for R_CT_ (Figure S4), which should increase as a function of film thickness, demonstrated a virtually flat trend at more dilute SAM concentrations, implying a porous SAM to redox reporters (MCH:Aptamer > 25:1) but demonstrated a substantial increase at high concentration SAMs inferring occlusion (no MCH or a 1:1 ratio). Error bars for the baseline values are larger than that expected solely based on SAM thickness but can be explained by simple electrode geometry effects, which is why all sensor outputs were reported as the normalized change in R_CT_. 

**Figure 3:**
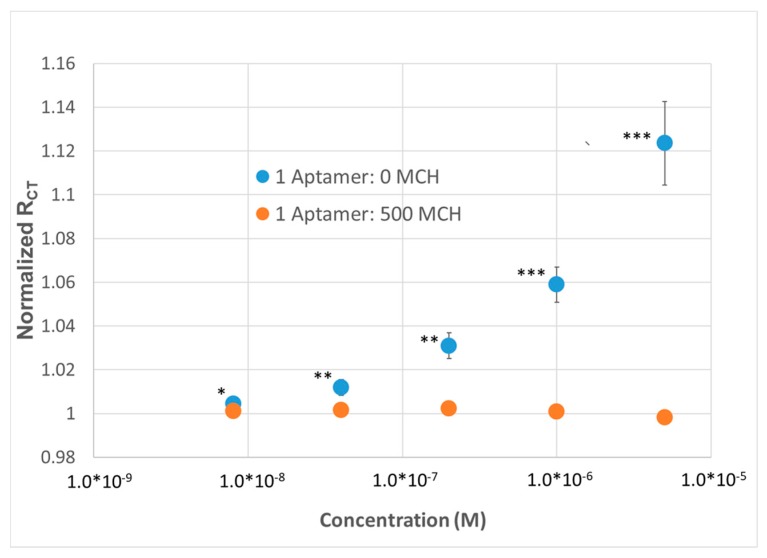
Demonstration of lysozyme aptasensor (n = 4) using EIS using a dense, thick SAM (1:Aptamer: 0 MCH) and a diffuse SAM (1 Aptamer: 500 MCH). *p* values < 0.05 (*), 0.01 (**) and 0.001 (***) determine using a two-tail *t*-test.

Multiplexed sensing was achieved using a custom jig (see Methods, Figure S2) that enables near-simultaneous testing of up to 6 electrodes within the same solution. We tested the response of the electrochemical sensors to lysozyme across 5 concentrations in 1×HBS containing a ferricyanide reporter tag. After fitting to a modified Randles cell (see Methods), we observed that a substantial change in signal was only observed for the highest density SAM (i.e., no dilution using MCH), implying that—(1) the aptamer was able to effectively bind its substrate at high packing densities and (2) the high packing density was necessary to enable a significant enough change in surface thickness to observe a substantial (R_CT_ > 2%) response (Figure 2B). To demonstrate the repeatability of the sensor, the sensor was run in multiplex (n = 4) and compared to a dilute SAM during the same titration. At a concentration as low as 8 nM, a statistically significant change in R_CT_ could be observed; at higher concentrations, up to a 12% change in R_CT_ could be observed with a 15% standard deviation, versus the SAM containing 500 MCH—1 aptamer (i.e., non-specific binding), which demonstrated no response (Figure 3). 

What is also of note is that the estimated limit of detection (~8 nM) is higher than the 3 nM dissociation constant reported in the literature, which should correlate with the concentration at which we observe ~50% sensor response. However, this can be explained by a multitude of factors including—(1) Changes in buffer conditions from 25 mM trizma, 192 mM glycine, 5 mM potassium phosphate, pH 8.3 [34] to 1 × HBS, pH 7.4, (2) depletion of molecules in solution upon binding to the lysozyme aptamer SAM and thus creating a disparity between the added concentration of target and the actual concentration of target; and/or (3) changes in affinity of the lysozyme BRE upon immobilization. 

### 3.2. Comparison of SWV versus EIS for Lysozyme Sensing

**Figure 4:**
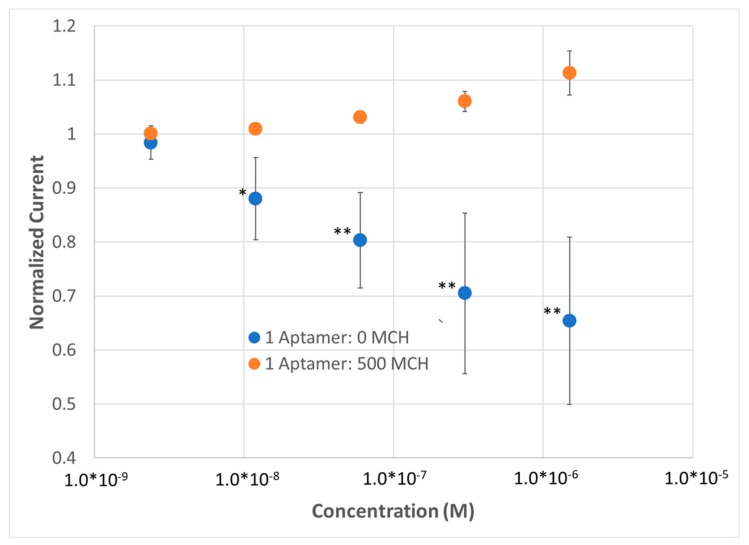
Demonstration of lysozyme aptasensor (n = 5) using square wave voltammetry (SWV) with a dense SAM versus a dilute SAM. *p* values < 0.05 (*), 0.01 (**) and 0.001 (***) determined using a two-tail *t*-test.

EIS, while more informative due to its ability to measure both real and imaginary impedance at numerous frequencies, is costly in terms of circuitry and time. In order to determine if a single frequency measurement could replace EIS, we explored SWV. SWV has the benefit of maintaining a single frequency and simply sweeping potential to create a peak in current that corresponds to the redox potential of the given system. In order to pick a frequency that would best reflect on R_CT_, a low frequency of 10 Hz, corresponding roughly to R_Soln_ + R_CT_ on the EIS plot, was chosen. The normalized peak current was measured instead of R_CT_ for EIS and plotted versus concentration of analyte in the 1×HBS containing the ferricyanide redox reporter. The result supported the use of EIS when practical (Figure 4)—(1) A lower limit of detection (LLOD) was now observed for the high-density BRE sensor (sub-24 nM for SWV compared to 8 nM for EIS) that is higher than using EIS according to statistical analysis. (2) The SAMs with low BRE density were now showing an opposite response to lysozyme compared to SAMs with high BRE density (increase in current versus decrease in current), likely corresponding to a shift in the observed solution resistance that is no longer accounted for and removed when fitting the Randles circuit for EIS measurements. (3) The observed variability was substantially greater for SWV compared to EIS (SD of ~33% for SWV vs. ~10% for EIS). Regardless, the efficacy of replacing EIS with SWV for sensing was demonstrated and this platform was used moving forward to probe SAMs that might be more amenable to reducing non-specific binding.

### 3.3. SAM Blocking Layers and Impact on Sensing

**Figure 5:**
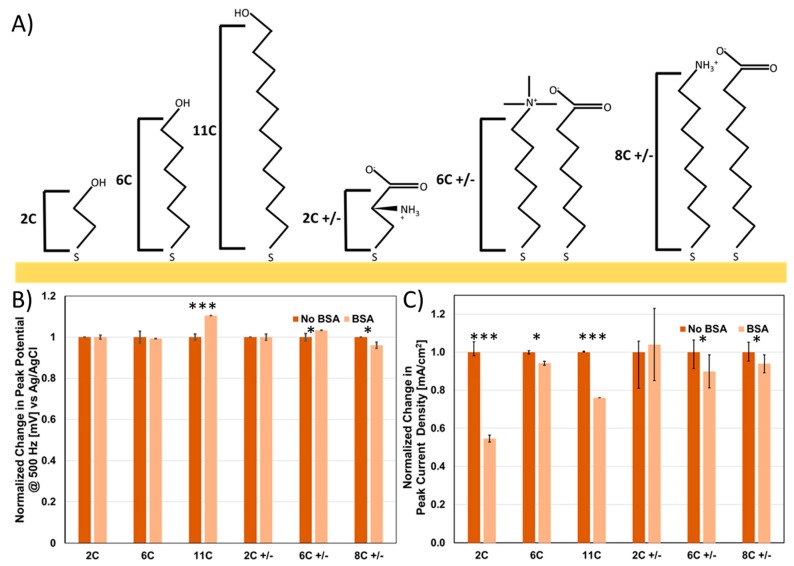
For SAMs of different composition (**A**), a normalized comparison of peak potential (**B**) and peak current (**C**) pre and post addition of the fouling agent bovine serum albumin (BSA) to test resistance to non-specific binding. *p* values < 0.05 (*), 0.01 (**) and 0.001 (***) determined using a two-tail *t*-test (n = 4).

SAM selection for sensors is imperative to performance, as the SAM has the function to both passivate the sensor surface as well as to prevent non-specific binding of analytes to gold. For example, there is a “Goldilocks” type paradigm with regards to SAM thickness; too thin of a SAM will result in spontaneous desorption, while too thick of a SAM will result in an increased R_CT_ and thus a substantial shift in the observed peak potential [44,45]. In order to probe the effects of SAMs on impedance sensing, we tested a variety of representative SAMs to probe their impact on (1) passivation, (2) voltage drop and (3) non-specific binding. To test the impact of chain length, we used the following hydroxy-terminated SAMs, 2-mercaptoethanol (2C), 6-mercaptohexanol (6C) and 11-mercaptoundecanol (11C) (Figure 5A). Anti-fouling SAMs have been tested for electrochemical sensing, primarily either zwitterionic SAMs or pegylated SAMs. In both cases, the hydrophilicity of the groups on the surface attracts a thin surface water layer, preventing hydrophobic absorption and thus non-specific fouling. Therefore, we also tested analogous SAMs with similar chain lengths that were zwitterionic—cysteine (2C +/−), MTAB mixed with mercpatohexanoic acid (6C +/−) and 8-mercaptooctanoic acid mixed with 8-amino-1-octanethiol (8C +/−) (Figure 5A). 

Within that subset, we focus our discussion on the electrochemistry of a non-traditional SAM molecule, MTAB. This molecule retains a positive charge (choline head-group), enabling the deposition of zwitterionic SAMs, while also being unreactive with amine-linker chemistry, such as succinimidyl esters, that are used to immobilize antibodies and other proteins through reacting with the lysine sidechain. 

The peak potential (Figure 5B) (cAg/AgCl) and the peak current (Figure 5C) were measured both pre and post fouling with BSA using 1×HBS doped with a ferricyanide reporter molecule. As expected, a substantial drop in peak current was observed as a function of chain length (2C < 6C < 11C) for the hydroxyl terminated SAMs (Figure S5B). What was interesting was that in comparison, the same current was observed for 2C +/− and 8C +/− but not for 6C +/− (Figure S5B). The counterintuitive disparity between 8C +/− and 6C +/− can be explained by the difference in their respective head-groups (amino group and choline group respectively). The two main differences in these head groups is that (1) amino groups have labile protons while choline groups (quaternary amines), contain no labile groups, (2) the methyl groups attached to the choline help to sterically occlude the positive charge on the amine. We hypothesize that either the reduction in charge density for the choline group or the inability to conduct protons explains this difference, which is a key consideration for SAM selection. 

Peak potential (voltage where maximum current is observed, Figure S6C) revealed a similar trend to the peak current, indicating that the lower current is a result of passivation and thus indicative of a voltage drop, and thus a peak observed at a more oxidative voltage. More specifically, we observed an increasing shift in the observed SWV peak with greater passivation of the surface, from 250 mV (C2) to 380 mV (C6) to 680 mV (C11) at 500 Hz (vs. Ag/AgCl). In contrast for the zwitterionic SAMs, cysteine and C8 +/− had virtually the same peak potential as C2, while C6 +/− had a peak potential slightly greater than C6, again supporting that eliminating the lability of the SAM headgroups creates a more resistive pathway and thus a voltage drop that must be accounted for in experimental design. In contrast, cysteine and C8 +/− have very similar responses; we believe this is readily explained through the capacitor created on the surface of the electrode, enabling rapid electron transfer through the conductive ion network. This is supported by EIS measurements for zwitterionic SAMs (Figure S7), as they have an extremely low R_CT_ that cannot be readily separated from non-Faradic circuit elements, confirming previous work where addition of charge density to the SAM resulted in a reduction of R_CT_ [46,47].

To further identify which of these SAMs were suitable for minimizing non-specific binding, we determined the change in current and peak potential upon addition of 100 µM BSA. Unsurprisingly, we observe the greatest decrease in current for 2C (instability) and 11C (low hydrophilicity) (Figure 5B), with, in some cases, a corresponding voltage shift as a result of non-specific binding (Figure 5C). 6C, 6C +/−, 8C +/− and 2C +/− in comparison all are minimally perturbed by addition of BSA (Figure 5B,C), demonstrating their suitability for deployment in electrochemical sensors as well as the compatibility of zwitterionic SAMs as anti-fouling components. 

### 3.4. SWV and Zwitterionic SAMs

**Figure 6:**
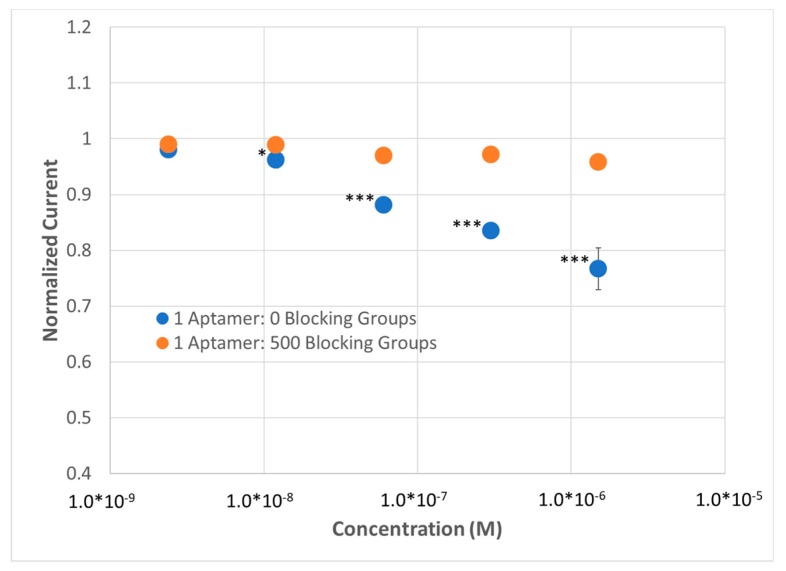
Demonstration of lysozyme aptasensor (n = 4) with a 6-carbon zwitterionic SAM (C6 +/−) measured using SWV at 10 Hz in 1 × HBS containing 10 mM ferricyanide redox reporter. *p* values < 0.05 (*), 0.01 (**) and 0.001 (***) determined using a two-tail *t*-test.

As discussed in the previous section, zwitterionic SAMs are used as anti-fouling layers for electrochemical sensing [28]. Another alternative to a phosphocholine group is to use alternating positively charged and negatively charged groups [45]. Therefore, to demonstrate the utility of zwitterions in aptasensors, we tested the model lysozyme/lysozyme aptamer system using SWV; EIS was not tested due to the inability to fit an expected Randles circuit to observed EIS spectra (Figure S7). Similar to what was observed with MCH SAMs, we observed a substantial difference in peak current at as low as 24 nM and a surprisingly consistent sensor performance, indicating the compatibility of SWV with zwitterionic SAMs (Figure 6). In comparison to the MCH SAM, we observed a decrease in response magnitude but an increase in reproducibility using the zwitterionic SAM containing a high density of BRE; we also observed a substantial decrease in the magnitude of response from the dilute SAM, potentially as a result of eliminating non-specific binding. The choice of frequency, as one would expect, was critical to obtaining the optimal sensor response. For example, monitoring SWV peak current at 100 Hz yielded no statistically significant difference between the two different SAMs (data not shown) but that same sensor demonstrated a significant, robust response in peak current at 10 Hz (Figure 6). 

### 3.5. SWV, Zwitterionic SAMs and Methylene Blue

One of the disadvantages with ferricyanide as a redox reporter is that its redox potential is oxidative (~0.4 V vs. SHE), and thus may damage the SAM during extended sensing. Additionally, it eliminates the ability to use thicker SAMs that might be more stable but would place the electrochemical window of detection at a voltage which would result in substantial background water oxidation and/or SAM oxidation. In addition, it has been demonstrated that ferricyanide can release cyanides that will etch gold over-time [31]. Therefore, we tested the use of methylene blue in aqueous solution as the redox reporter. 

Most commonly, methylene blue has been covalently linked to aptamers creating electrochemical-aptamer based sensors [19]. The robustness of the redox reporter over repeated cycling, as well as the near-ideal redox potential (~0.0 V vs. SHE), made it worth exploring as a redox reporter in solution. Drawbacks are that methylene blue tends to intercalate into hydrophobic pockets (logP 2.61), as well as into the double helix of DNA [32]. Regardless, a positive result would demonstrate the utility of such an approach in select circumstances to allow for measurements to be obtained at potentials near 0 V (vs. SHE). 

**Figure 7:**
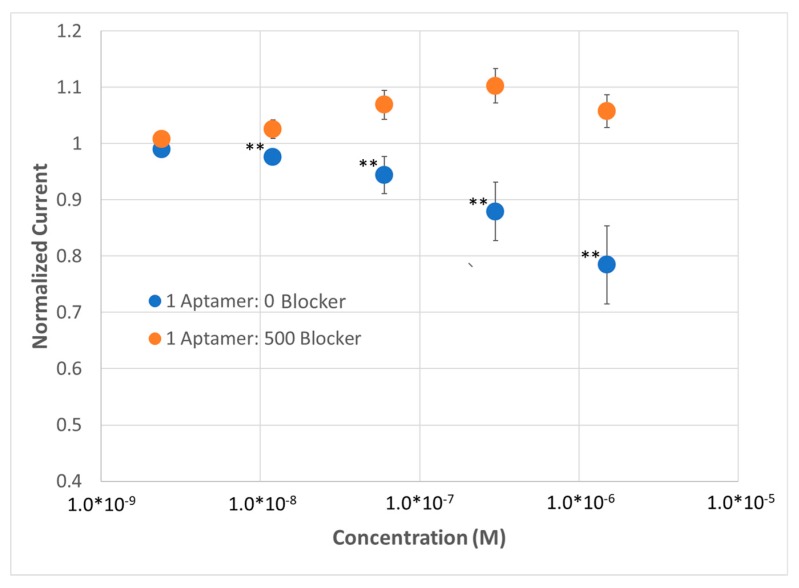
Demonstration of lysozyme aptasensor (n = 3) with a 6-carbon zwitterionic SAM using SWV at 10 Hz and a 100 µM methylene blue redox reporter. *p* values < 0.05 (*), 0.01 (**) and 0.001 (***) determined using a two-tail *t*-test.

Attempts to use EIS yielded Nyquist plots with apparently low values of R_CT_ and that are indicative of diffusion limited electrochemical reactions (Figure S8). Therefore, we decided again to probe the redox reporter using SWV with the 6C+/− SAM. A highly similar sensor response was observed for SAMs with a high aptamer concentration compared to sensors tested with ferricyanide as a redox reporter. However, a substantially greater background change in current (increase in current as a function of titration for the dilute SAM), as well as inconsistency in the measurement, as reported by the standard deviations observed were present when methylene blue was the redox reporter used in the experiment. Regardless, this result still demonstrated the utility of methylene blue as a potential redox reporter tag in solution if the sensor needs to be operated at a lower potential (Figure 7).

## 4. Discussion

Impedance based biosensors have become commonplace in the literature for proof-of-concept studies, with fewer of those investigating the interplay between SAM density and sensor performance. While those manuscripts have used the addition of blocking groups to decrease BRE density, we have performed the first characterization of the thicknesses of these mixed SAMs and how blocking group density impacts SAM thickness. Most notably, in the literature, researchers have for the most part neglected to look at SAMs that contain a low ratio (i.e., 1:1 to 1:5) blocking groups per BRE [22]; however, it is clear from the data that there is an exponential decay in SAM thickness as the number of blocking groups per aptamer increases. Therefore, to probe the impact of SAM thickness, it is most important to use a sampling algorithm that is more based on a geometric series (i.e., 1, 3, 9…) than on a simple arithmetic series (i.e., 3, 6, 9…). As demonstrated, the impact of SAM density on sensor sensitivity can be significant and therefore is a critical element to array for impedance based sensors. 

The SAMs derived from this measurement can then be applied to both EIS sensing, as well as SWV sensing at a select frequency. With EIS measurements, we are reporting impedance, which includes both the real (i.e., resistance) and imaginary components. For SWV we are reporting the current, which according to V=IR, is simply the inverse of resistance. Therefore, we would expect for a sensor where EIS impedance increases upon analyte binding, the current decreases; as expected, we observed this trend at the higher aptamer density where we observed maximum sensor response. For SWV measurements, we found it most useful to probe lower frequencies (< 10 Hz); the impedance observed at this frequency should, according to the modified Randle’s cell, account for the full resistance to charge transfer, as well as some additional resistance due to localized depletion of the redox reporter (diffusion effects). 

Various SAMs have been used in impedance-based biosensing; yet the interplay between changing the SAM components and electrochemical sensing had not yet been fully characterized. Since passivation of surfaces creates a resistance, surface passivation is expected to cause (1) a reduction in current and (2) an increase in the observed peak potential in the SWV, corresponding to the electrochemical potential in the particular system. As demonstrated, SAM thickness for uncharged, hydrophilic head-groups seems to follow the predicted pattern, where thicker SAMs correlate to a reduction in current and an increased in the observed redox peak. In contrast, by adding a zwitterion onto the surface, originally added to help prevent non-specific binding, we observe no impact of SAM thickness on impedance. However, moving from an amine group to a choline group may prevent proton conduction since labile protons (H_3_O^+^ + R-NH_2_ ←→ R-NH_3_^+^, pKa ~10.5) are replaced by stable methyl groups that form a quaternary amine complex and thus increase resistivity. Regardless, in all cases, the zwitterionic SAM reduced R_CT_ to the point that implementation of EIS was difficult. Clearly, it is important when doing SAM design and implementation to test the impact on the redox probe used as well as the electrochemical experiment to be selected. 

To demonstrate the efficacy of the anti-fouling six-carbon zwitterionic SAM, we then performed SWV using both a ferricyanide and a methylene blue reporter. While the ferricyanide redox reporter demonstrated a more ideal response, methylene blue also worked as a redox reporter and suggests that future exploration of redox reporters with redox potentials closer to 0 V vs. SHE and with various properties may be useful to optimize these types of impedance sensors.

## 5. Conclusions

This manuscript demonstrates the impact that select parameters (Table 1) can have on impedance based biosensing. Most relevantly, optimizing SAM density, as well as the interplay between electrochemical detection mechanism, SAM selection and redox reporter, are critical to development of high-performing sensors. While this work highlights how several of these factors impact impedance-based sensors, using a lysozyme aptasensor as a model, the key parameters studied here can be extended to other novel biosensing targets and methods to achieve enhanced sensing sensitivity and selectivity. Future work needs to be done to apply this methodology to other BREs, such as peptides and nanobodies, as well as to probe reagentless sensing platforms to further define the parameters that need to be considered for all impedance-based sensors. Understanding these key principles will enable more focused and improved development of analyte-specific sensors to enable real-time biosensors. 

## Figures and Tables

**Table 1:** Summary table of experiments and findings on how biorecognition element (BRE) density, SAM Selection, Electrochemical Sensing Mechanism and Selection of Redox Reporter Impact Sensing.

Factors Explored	Method/Mechanism	Physiochemical Impact	Impact on Sensing
**Density of BRE**	BRE to Blocking Group Ratio	Ratio impacts SAM density, as observed by thickness.	Density of SAM directly impacts sensor sensitivity (change in R_CT_ as function of analyte concentration).
**SAM Chain Length**	Selection of carbon chain between thiol and hydrophilic head-group	Long chains passivate SAMs while short chains are labile.	Longer chains have greater resistance to charge transfer. Shorter chains are more prone to fouling/stability issues.
**Sensing Mechanism**	Selection of either frequency scan (EIS) at a fixed potential or voltage scan (SWV) at a fixed frequency	SWV can be applied to minimize time/electronics demand. EIS is more informative.	SWV is ideal for rapid sensing and can be more widely implemented. However, SWV may miss critical information from EIS. SWV is more compatible with zwitterionic SAMs.
**SAM Charge Density**	Thiolated molecules with varying functional groups to change surface charge	Zwitterionic surfaces attract water preferentially, limiting hydrophobic fouling.	Zwitterion has been demonstrated to reduce fouling but adds capacitor to surface.
**Redox Reporter**	In-solution reporter probes thickness of SAM layer on surface (resistance/impedance)	More spontaneous redox reporters (~0 V vs. SHE) reduce required energy and increases current, preventing SAM oxidation.	Methylene blue is more stable but is pH sensitive and hydrophobic, limiting applications.

## Supplementary Materials

The following are available online at https://www.mdpi.com/1424-8220/20/8/2246/s1, Figure S1: MTAB Synthesis, Figure S2: Custom Multiplexed Electrochemical Jig, Figure S3: Modified Randle’s Cell Used for Impedance Fitting, Figure S4: Representative Square Wave Voltammograms at 10 Hz for Various SAMsFigure S5: Resistance to Charge Transfer vs. Aptamer Density, Figure S6: Impact of SAM on EIS, Figure S7: Impact of SAM on EIS, Figure S8: Impact of Redox Reporter on EIS.
