# Supplementary material for: Impact of Self-Assembled Monolayer Design and Electrochemical Factors on Impedance-Based Biosensing
Source: Sensors (Basel). 2020 Apr 16;20(8):2246. doi: 10.3390/s20082246 (PMC7218866; doi:10.3390/s20082246)
Supplement: Supplementary file 1 [file sensors-20-02246-s001.pdf]

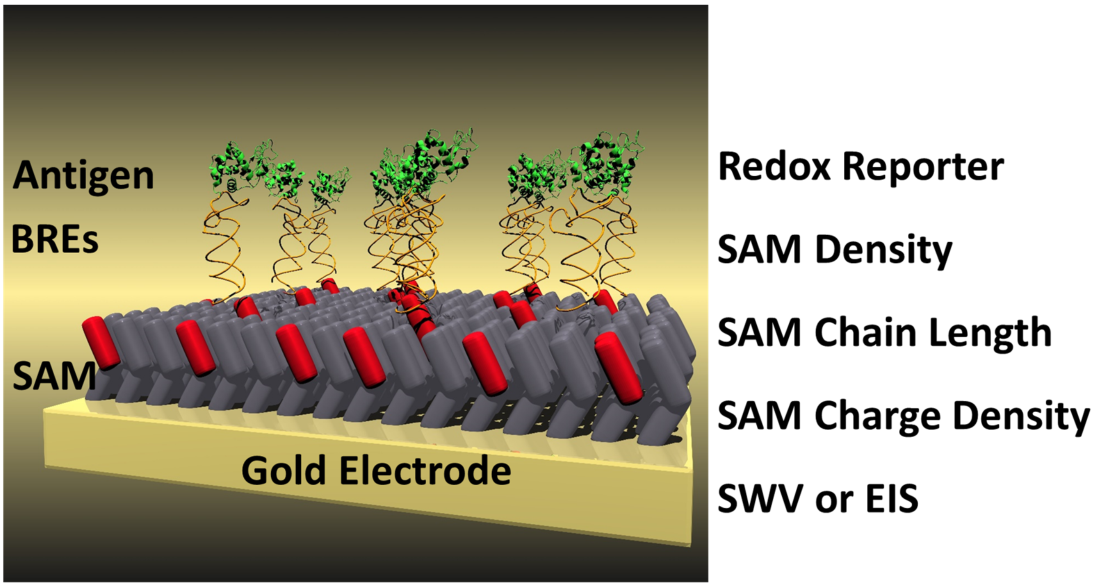

Table of Contents Graphic. Impedance sensors comprised of blocking groups and BREs to form a heterogenous SAM can be generated on gold electrodes to detect analytes of interest; sensing performance is influenced by the redox reporter, SAM density, SAM chain length, SAM charge density electrochemical technique (SWV or EIS) selected.

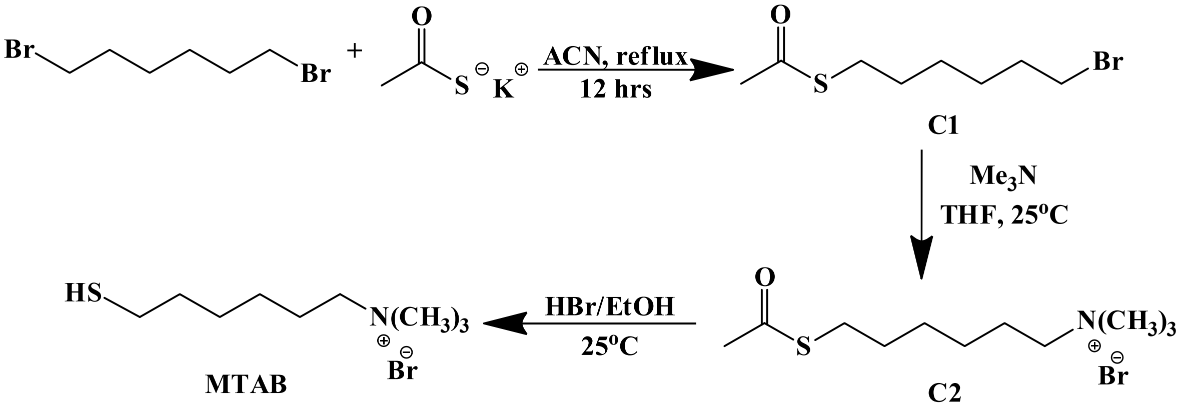

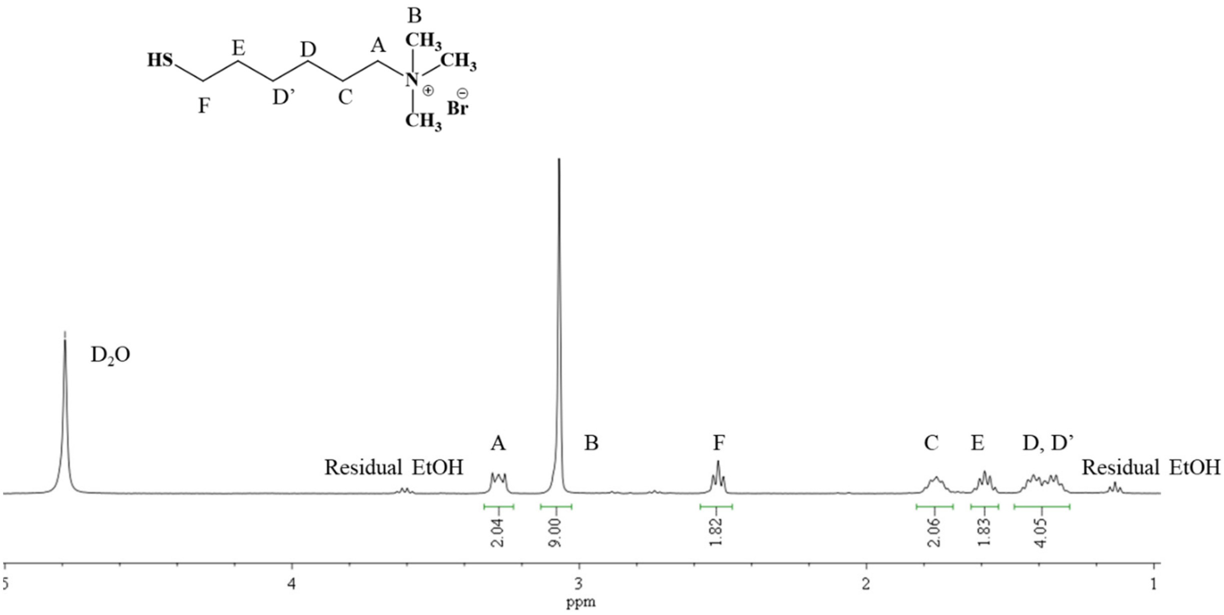

SI Figure 1. *MTAB Synthesis*: Synthesis scheme (top) and NMR spectra of final product (bottom) for synthesis of MTAB.

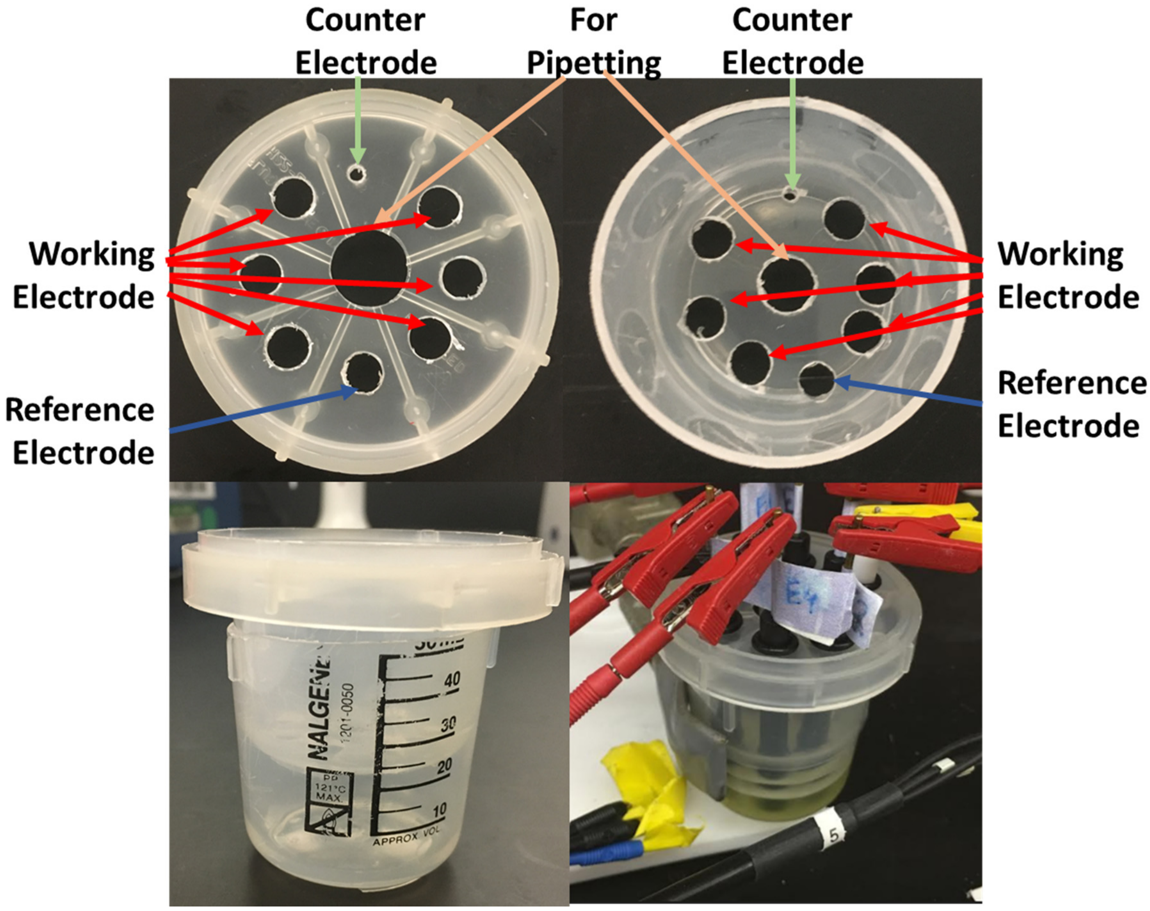

SI Figure 2. *Custom Multiplexed Electrochemical Jig*: Custom three-component jig created through drilling electrode-size holes in a polypropylene top-plate (**A**) and a 50 mL Nalgene beaker with spout removed (**B**) placed inside a third 50 mL beaker that serves as a reservoir (**C**). This set-up enables multiplexed sensing for up to six gold electrodes (**D**) for impedimetric sensing in the same reservoir with a common reference and a common counter electrode and enables both titrations as well as mixing via pipette.

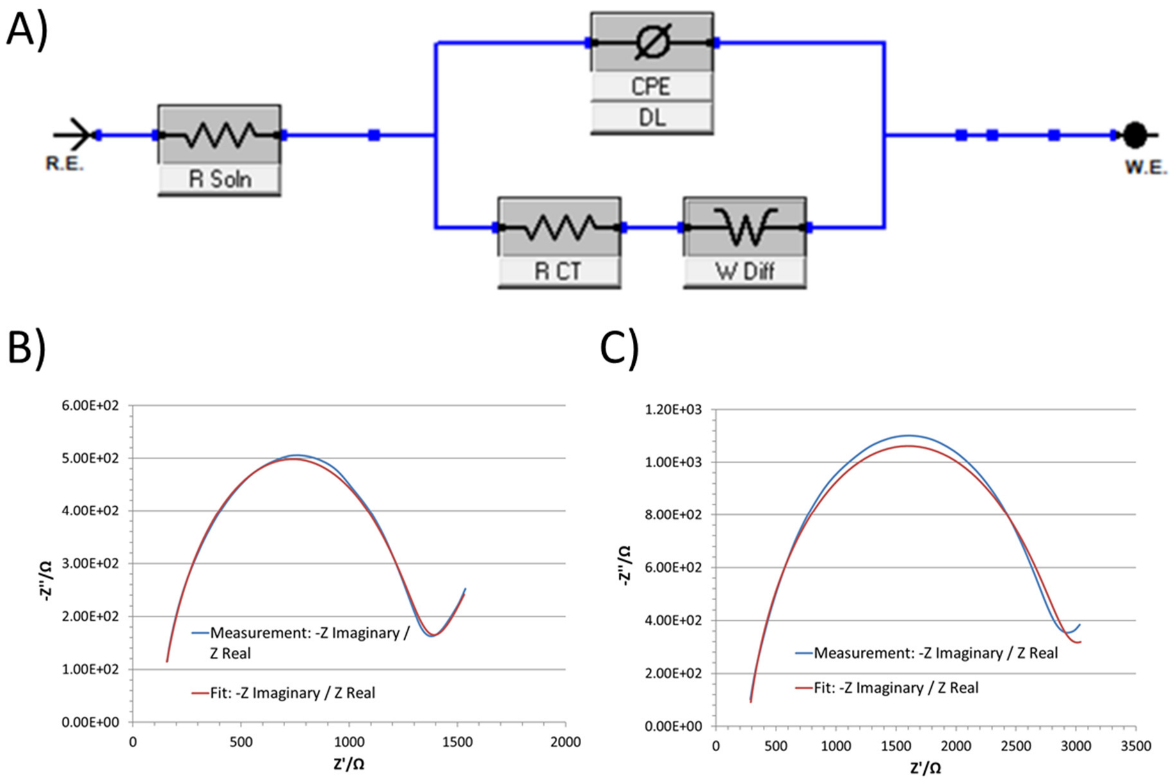

SI Figure 3. *Modified Randle's Cell Used for Impedance Fitting:* A modified Randle's Cell (**A**) used for fitting the pseudo-circuit for the electrochemical cell (R.E represents gold working electrode, W.E. represents platinum counter electrode) where the solution resistance (R Soln) is probed at high frequencies, a constant phase element to account for the imperfect capacitance from the double layer (CPE, DL), a resistance to charge transfer (R CT), as well as a Warburg diffusion element for mass-limited reactions at ultra-low frequencies. Examples of the model fit to a low-density aptamer SAM (**B**) and a high-density aptamer SAM (**C**) are demonstrated.

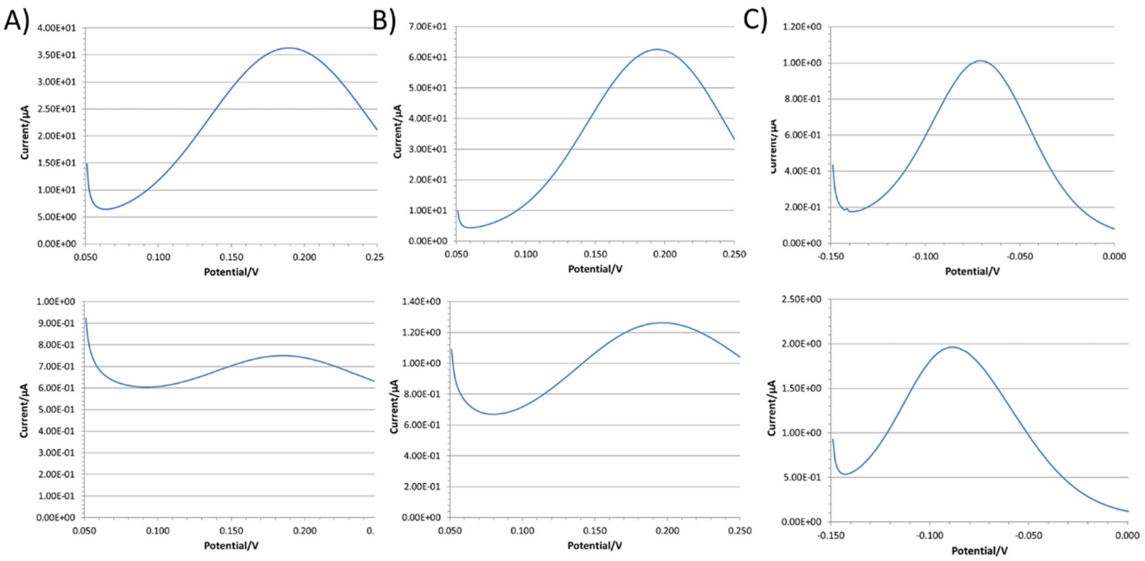

SI Figure 4. *Representative Square Wave Voltammograms at 10 Hz for Various SAMs:* Representative square wave voltammograms for both low density (top) and high density (bottom) aptamer SAMs with MCH SAM using a ferricyanide redox reporter (**A**), 6C +/- using a ferricyanide redox reporter (**B**) and 6C +/− using a ferricyanide redox reporter (**C**).

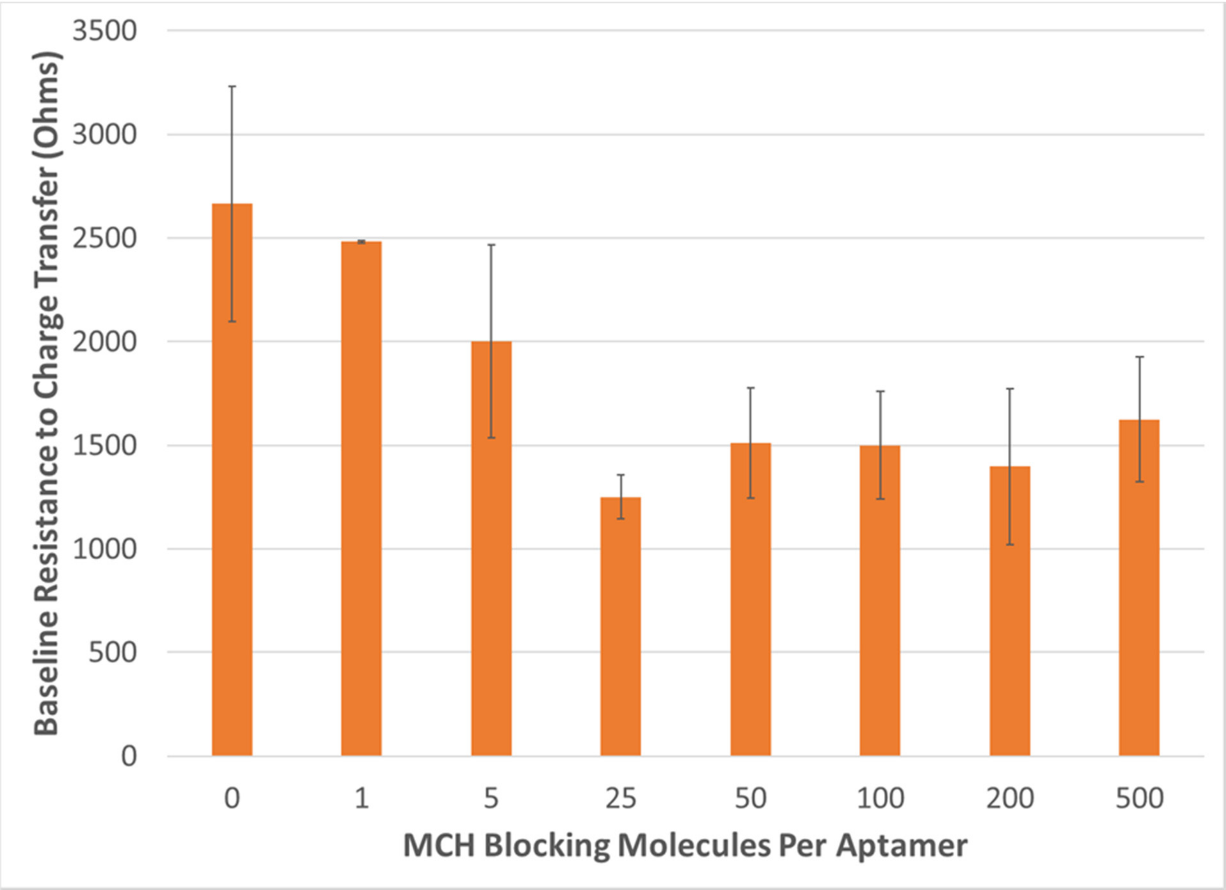

SI Figure 5. *Resistance to Charge Transfer vs Aptamer Density:* Baseline (SAM with no analyte added) resistance to charge transfer values determined by EIS as a function of MCH:Aptamer ratio.

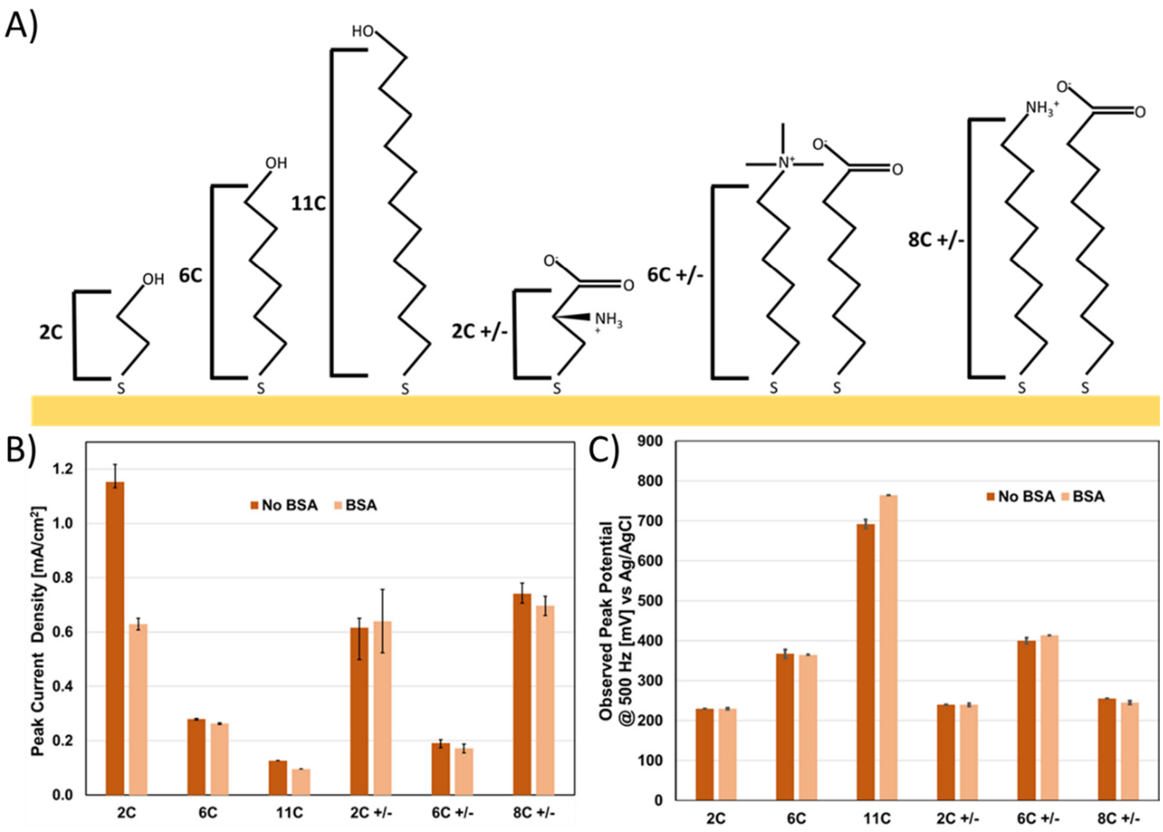

SI Figure 6. *Impact of SAM on SWV and anti-fouling properties*: Depiction of SAMs demonstrating unique characteristics (**A**). SWV measurements using Gamry (n = 4) demonstrates that current is a function of SAM chain length (2C < 6C < 11C), that zwitterionic SAMS have higher current regardless of chain-length (2C +/− vs 8C +/−) (**B**) and that the addition of a choline head-group makes the SAM substantially more resistive (6C +/−) (**C**). The peak potential follows a similar pattern, where peak current is inversely proportional to peak potential (**B** & **C**).

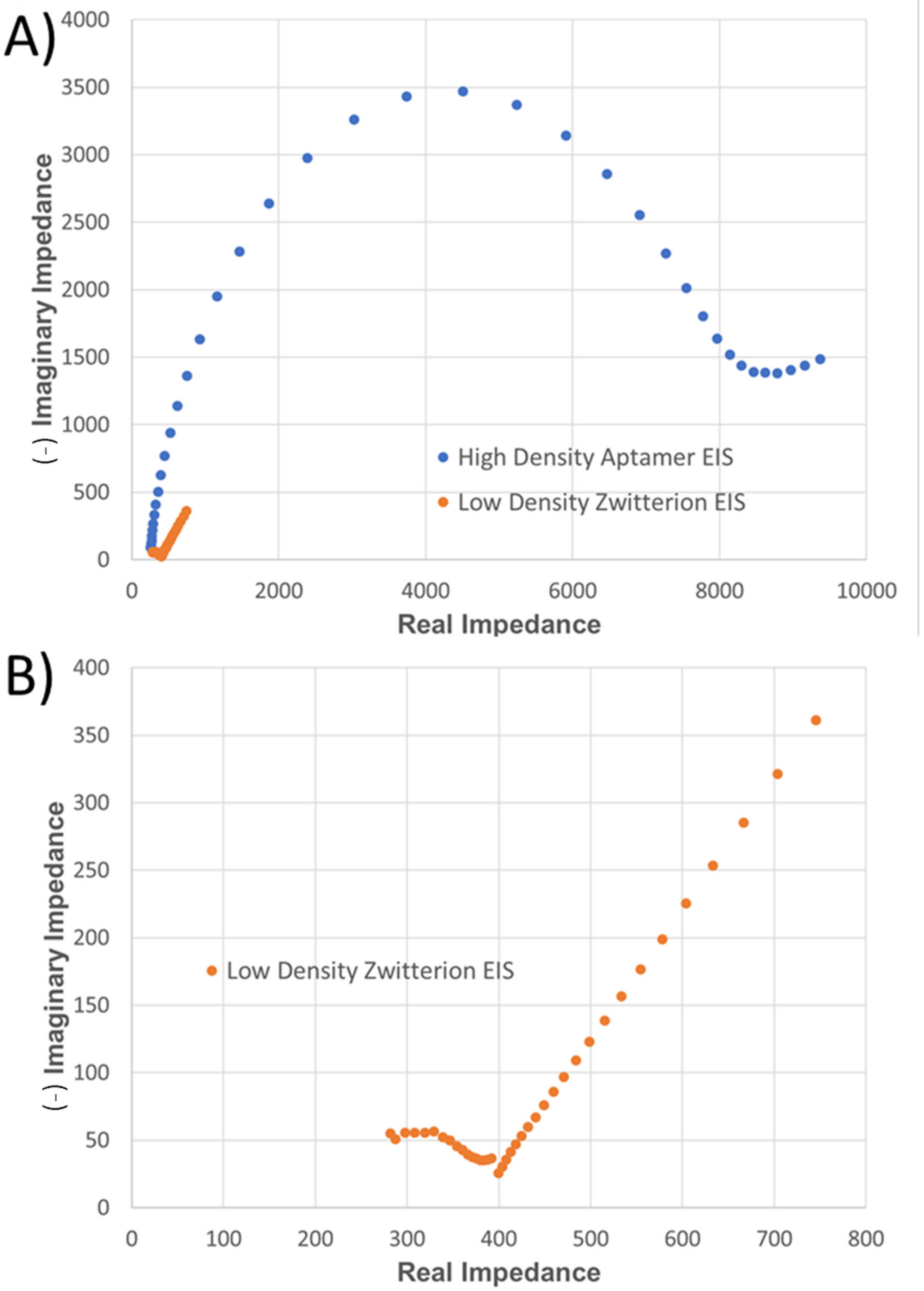

SI Figure 7. *Impact of SAM on EIS*: Overlay (**A**) of a zwitterionic SAM with high aptamer density and low aptamer illustrates the drastic change observed in resistance to charge transfer. An expanded plot of just the low-density zwitterion Nyquist plot (**B**) demonstrates the difficulties obtaining EIS on zwitterionic surfaces, due to the minimal increase in imaginary impedance over the time constant associated with the double layer capacitance. EIS measurements were acquired using a PalmSens3 Potentiostat.

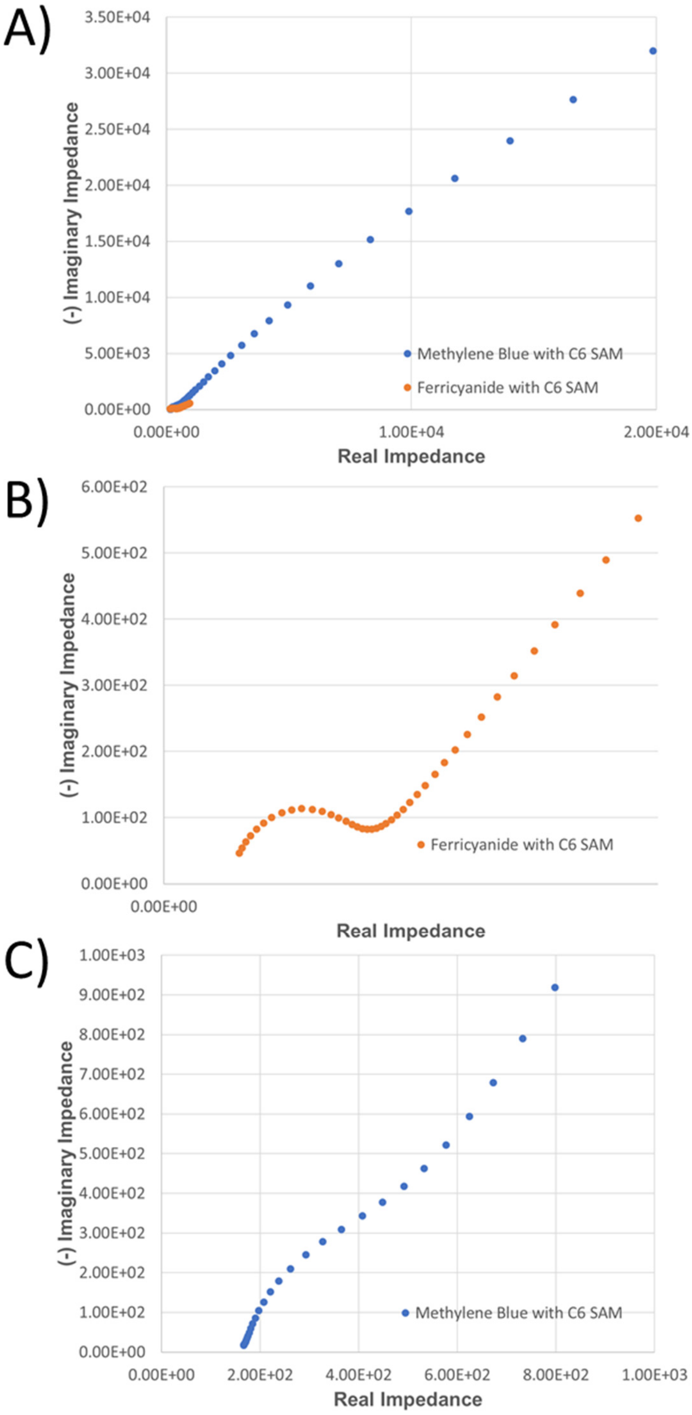

SI Figure 8: *Impact of redox reporter on EIS*: Overlay (**A**) of Nyquist plots from EIS measurements obtained using a MCH SAM with both ferricyanide (**B**) and methylene blue (**C**) redox reporters. Plots B and C were expanded in order to focus in on the region corresponding to the time constant corresponding to the double layer capacitance in the modified Randle's cell. These plots demonstrate that while a typical Randle's cell is observed for ferricyanide (**B**), methylene blue (**C**) results in Warburg diffusion being the primary observed feature, minimizing the utility of EIS when methylene blue is used as the redox reporter. EIS measurements were acquired using a Gamry 600 Potentiostat.
